# Ethnic differences in the association between cardiovascular risk factors and psychological distress in a population study in the Netherlands

**DOI:** 10.1186/1471-2458-12-1090

**Published:** 2012-12-18

**Authors:** Agnes C Schrier, Joanne K Ujcic-Voortman, Matty AS de Wit, Arnoud P Verhoeff, Ralph Kupka, Jack Dekker, Aartjan TF Beekman

**Affiliations:** 1 Altrecht Institute for Mental Health Care, Utrecht, The Netherlands; 2 Department of Epidemiology, Public Health Service Amsterdam, Documentation and Health Promotion, Amsterdam, The Netherlands; 3 Department of Sociology and Anthropology, University of Amsterdam, Amsterdam, the Netherlands; 4 Department of Psychiatry, VU University Medical Center, Amsterdam, The Netherlands; 5 Department of Clinical Psychology, VU University Medical Center, Amsterdam, The Netherlands; 6 VU University Medical Center, EMGO Institute for Health and Care Research, Amsterdam, The Netherlands

**Keywords:** Obesity, Cardiovascular, Depression, Ethnicity, Epidemiology

## Abstract

**Background:**

There is growing body of evidence of an association between cardiovascular risk factors and depressive and anxiety symptoms. The purpose of this study was to investigate whether these associations are similar in ethnic minority groups.

**Methods:**

A random urban population sample, aged 18+, stratified by ethnicity (484 native Dutch subjects, 383 Turkish-Dutch subjects, and 316 Moroccan-Dutch subjects), in Amsterdam, the Netherlands, was interviewed with the Kessler Psychological Distress scale (K10) in combination with measurements of several cardiovascular risk factors. The association of psychological distress (defined as a K10 score above cut-off of 20) with cardiovascular risk factors (obesity, abdominal obesity, hypertension, hypercholesterolemia, low HDL cholesterol levels or diabetes), ethnicity and their interaction was analyzed using logistic regression analyses, stratified by gender and adjusted for age.

**Results:**

Cardiovascular risk factors were not significantly associated with psychological distress in any of the gender/ethnic groups, with the exception of a positive association of obesity and hypertension with psychological distress in native Dutch women and a negative association of hypertension and psychological distress in Turkish men. Interaction terms of cardiovascular risk factors and ethnicity were approaching significance only in the association of obesity with the K10 in women.

**Conclusion:**

In this cross-sectional multi-ethnic adult population sample the majority of the investigated cardiovascular risk factors were not associated with psychological distress. The association of obesity with psychological distress varies by gender and ethnicity. Our findings indicate that the prevention of obesity and psychological distress calls for an integrated approach in native Dutch women, but not necessarily in Turkish-Dutch and Moroccan-Dutch women, in whom these problems may be targeted separately.

## Background

The high co-occurrence of depressive illness and cardiovascular disease has stimulated research into the factors linking these two conditions. Numerous studies have shown that several cardiovascular risk factors are associated with depressive and/or anxiety symptoms [1,2]. In particular the findings from longitudinal studies showing that waist circumference predisposes to depression and vice versa, have lead to the suggestion that specific pathophysiological mechanisms link depression and visceral fat accumulation [3,4]. In cross-sectional studies lipid disorders (total cholesterol, low-density lipoprotein (LDL) cholesterol, high-density lipoprotein (HDL) cholesterol and high triglyceride levels) were found to be associated with depressive disorder subtypes, whereas hypertension or hyperglycemia were not or only rarely [5-7]. However, these associations may be confined to women [7-10]. Interestingly, subgroup analyses have revealed race/ethnic differences in the association between cardiovascular risk factors and depression, because studies report a stronger association between obesity and depression in whites than in blacks or Hispanics in the US population [11-13]. This applies not only to obesity, but also to the metabolic syndrome, a constellation of cardiovascular risk factors of metabolic origin: depressive symptoms increase the odds of metabolic syndrome in white, but not in black, individuals [14]. These findings, however, are not unequivocal and the background of these findings is still largely unclear [15]. Studies with a broader range of race/ethnicity may help to clarify the contribution of social and cultural influences to the relationship between cardiovascular risk factors and mood or anxiety syndromes.

Labor migration from the 1960s onward brought large numbers of migrants from Turkey and Morocco to Europe, and studies have reported a higher prevalence of depressive and anxiety disorders or symptoms among Turkish and Moroccan immigrants than among native citizens [16,17]. These migrant groups also have a high risk of developing some, but not all, cardiovascular risk factors. In a population study in Amsterdam we showed that obesity and abdominal obesity are far more common among Turkish-Dutch and Moroccan-Dutch women than among native Dutch women [18]. Hypertension was less common in Turkish-Dutch and Moroccan-Dutch citizens of Amsterdam than among native Dutch citizens [19]. In these Turkish and Moroccan groups total cholesterol levels were low, as well as HDL cholesterol levels, resulting in an unfavourable total/HDL cholesterol ratio in particularly the Turkish ethnic group [20]. Diabetes was more common among Turkish-Dutch and Moroccan-Dutch than among native Dutch citizens of Amsterdam [21].

In the present study, we investigated ethnic differences in the association between several cardiovascular risk factors and psychological distress in a population based-sample of native Dutch, Turkish-Dutch, and Moroccan-Dutch inhabitants of Amsterdam. As we anticipated gender differences in these associations, we studied men and women separately.

## Methods

The Amsterdam Health Monitor (AHM) is a cross-sectional population-based health survey, designed and conducted every 4 years by the Amsterdam Public Health Service. The AHM of 2004 was based on a random sample from the municipal population register, stratified by age (18–34 years, 35–44 years, 45–54 years, 55–64 years and 65 years and older) and ethnicity (1043 native Dutch, 913 Turkish-Dutch, 965 Moroccan-Dutch) [19]. Ethnicity was defined as Turkish-Dutch or Moroccan-Dutch by country of birth (first-generation immigrants) or country of birth of one or both parents (second-generation immigrants). Respondents whose parents were both born in the Netherlands were considered native Dutch subjects [22]. Selected residents were invited to participate in a health interview and examination at a local childcare center. The response rate was lower among men and among the youngest age groups. The final response rates were 46% in native Dutch, 50% in Turkish-Dutch and 39% in Moroccan-Dutch. All participants signed an informed consent form. The study protocol was approved by the Medical Ethics Committee of the Academic Medical Center, the University of Amsterdam, Amsterdam, the Netherlands.

A health interview was conducted in the respondent’s language of choice (Dutch, Turkish, Moroccan-Arabic, Berber, or English). The interviews were based on a structured questionnaire that was translated into Turkish, Arabic, and English, and validated via back-translation by certified translators. The interview included the Kessler Psychological Distress scale (K10), of which an official Dutch version is available [23-25]. The K10 is a useful diagnostic screening instrument in general population samples and is not biased with respect to gender and educational level. We previously found only minor differential item bias in relation to ethnic background [24]. The K10, a 10-item scale with total scores ranging from 10 to 50, is effective in detecting depressive and/or anxiety disorders when a cut-off point of 20 is used [26,27]. For the statistical analyses, missing K10 values were imputed (by replacement by individual mean item score), but only if respondents had answered at least 8 of the 10 K10 items. We defined psychological distress as a K10 score ≥ 20.

Information was collected about history of hypertension, hypercholesterolemia, diabetes, and relevant medication use; age; education in the country of origin and in the host country; and current employment status. A trained nurse examined the participants. Weight (adjusted for clothing by subtracting 1 kg) and height were recorded. Waist circumference was measured at the level midway between the lowest rib margin and the iliac crest; the measurement was performed in duplicate. Systolic and diastolic blood pressure were measured in duplicate in a seated position using a validated oscillometric automated device (Omron HEM-711), after a minimum of 5 minutes rest. Total cholesterol, HDL-cholesterol, glucose, and glycosylated hemoglobin (HbA_1c_) were measured in non-fasting blood samples, using standard laboratory techniques.

Obesity was defined as body mass index (BMI = weight/height^2^) ≥ 30.0 kg/m^2^[28]. Abdominal obesity was defined as waist circumference ≥ 94 cm for men and ≥ 80 cm for women [29]. Hypertension was defined as self-reported hypertension and current use of antihypertensive medication, or measured systolic blood pressure ≥ 140 mmHg or diastolic blood pressure ≥ 90 mmHg [30]. Hypercholesterolemia was defined as self-reported elevated cholesterol levels and current use of lipid-lowering medication, or measured non-fasting serum total cholesterol ≥ 6.5 mmol/L [30]. HDL cholesterol levels lower than 1.03 mmol/L in men and 1.29 mmol/L in women were defined as low [29]. Diabetes was defined as self-reported diabetes and use of oral hypoglycemic agents or insulin, or measured non-fasting glucose level ≥ 11.0 mmol/L combined with an HbA_1c_ level ≥ 48 mmol/mol (6.5%).

43 respondents had more than 2 missing items in the K10 and were excluded from analysis. In addition 103 respondents where excluded from whom an insufficient amount of blood was collected. We analyzed the data for men and women separately. Ethnic differences in sample characteristics, stratified by gender, were tested, using ANOVA for the continuous variables and chi-square tests for the dichotomous variables. Logistic regression analyses were conducted to assess the association between each of the cardiovascular risk factors and the presence of psychological distress, stratified by gender and adjusted for age. The final model included cardiovascular risk factor, ethnicity, interaction term of cardiovascular risk factor and ethnicity, and age. To control for confounding by socioeconomic status, we repeated these analyses with addition of educational level and employment status. All analyses were performed using SPSS 16.0.

## Results

The Turkish-Dutch and Moroccan-Dutch respondents, of which the vast majority were first-generation immigrants, had received little education and had high rates of unemployment (Table 1). They had higher levels of psychological distress, and obesity, abdominal obesity, low HDL cholesterol levels and diabetes were more common than in the native Dutch respondents. In contrast, hypertension, and hypercholesterolemia were more prevalent in the native Dutch group.

**Table 1:** Sample characteristics by gender and ethnicity

	**Men**			**Women**		
	**Dutch**	**Turkish**	**Moroccan**	**Dutch**	**Turkish**	**Moroccan**
	**(*n*** **= 201)**	**(*n*** **= 180)**	**(*n*** **= 175)**	**(*n*** **= 283)**	**(*n*** **= 203)**	**(*n*** **= 141)**
**Sociodemographic characteristics**						
Age (years (sd))	51.2 (14.9)	48.8 (12.8)	52.2 (13.4)	51.3 (15.4)	42.6 (13.8)	44.6 (13.8)*
Educational level (%)						
none or primary	12.1	57.5	63.9*	22.1	63.8	64.7*
secondary	87.9	42.5	36.1	77.9	36.2	35.3
Employment (%)						
employed^a^	63.6	40.2	37.0*	59.6	26.8	27.3*
housewife/man	1.0	2.3	1.2	6.5	37.4	51.5
retired	22.2	17.2	22.0	20.6	6.8	3.0
unemployed	13.1	40.2	39.9	13.0	28.9	18.2
**Psychological distress**						
K10 (mean (sd))	16.0 (5.9)	18.1 (8.1)	17.6 (9.1)*	16.0 (6.5)	18.6 (8.0)	17.9 (6.8)*
K10 ≥ 20 (%)	22.9	33.9	29.7	20.1	36.9	38.3*
**Cardiovascular risk factor prevalence (%)**						
Obesity	12.9	28.5	19.0 *	19.8	48.0	48.2 *
Abdominal obesity	56.2	72.2	68.0 *	75.3	87.6	85.0 *
Hypertension	58.9	52.2	46.7	47.1	35.7	33.6
Hypercholesterolemia	29.0	19.6	13.3 *	31.3	13.4	13.6 *
Low HDL cholesterol	14.9	37.2	30.9 *	19.4	53.2	42.6 *
Diabetes	5.1	11.0	18.8 *	2.9	8.4	16.1 *

Notes:

sd = standard deviation.

a) employed includes people with paid jobs and students.

* p < 0.05 (design-based chi-square or ANOVA) for difference between ethnic groups.

Overall, cardiovascular risk factors and psychological distress were not associated (Table 2). There were a few exceptions: In Dutch women, obesity and hypertension were associated with a greater likelihood of psychological distress (obesity, OR 2.52 (95% CI 1.31 - 4.86); hypertension, OR 2.85 (95% CI 1.44 – 5.64); both adjusted for age). In Turkish men, hypertension was associated with a lower likelihood of psychological distress (OR 0.44 (95% CI .022 – 0.90) adjusted for age). Only in the association of obesity with psychological distress in women the interaction term with ethnicity approached significance (p = .066). In all other analyses the interaction terms were not significant (p ≥ .10), indicating a similar relationship for all ethnic groups. Abdominal obesity, hypercholesterolemia, low HDL cholesterol levels and diabetes were not associated with psychological distress. Addition of the confounding variables educational level and employment status did not influence model outcomes (not presented in the table).

**Table 2:** **Cardiovascular risk factors as predictors of psychological distress (K10 ≥ 20), by gender and ethnicity**^**a)**^

	**Men**				**Women**			
		**OR**	**(95% CI)**	**p**		**OR**	**(95% CI)**	**p**
Obesity	Dutch	1.72	(0.69 – 4.28)	.25	**Dutch**	**2.52**	**(1.31 – 4.86)**	**.006**
	Turkish	1.35	(0.68 – 2.69)	.39	Turkish	1.07	(0.59 – 1.95)	.83
	Moroccan	0.86	(0.37 – 2.02)	.74	Moroccan	0.89	(0.44 - 1.78)	.74
Abdominal obesity	Dutch	0.79	(0.40 – 1.56)	.50	Dutch	1.16	(0.56 – 2.39)	.70
	Turkish	0.79	(0.39 – 1.63)	.53	Turkish	1.16	(0.46 – 2.90)	.75
	Moroccan	0.86	(0.43 – 1.75)	.68	Moroccan	0.92	(0.35 – 2.44)	.87
Hypertension	Dutch	1.11	(0.54 – 2.29)	.77	**Dutch**	**2.85**	**(1.44 – 5.64)**	**.003**
	**Turkish**	**0.44**	**(0.22 – 0.90)**	**.02**	Turkish	1.13	(0.58 – 2.22)	.72
	Moroccan	0.81	(0.41 – 1.62)	.55	Moroccan	1.41	(0.65 – 3.09)	.39
Hypercholesterolemia	Dutch	1.40	(0.68 – 2.87)	.37	Dutch	1.11	(0.58 – 2.13)	.74
	Turkish	0.25	(0.58 – 2.73)	.57	Turkish	0.54	(0.22 – 1.37)	.20
	Moroccan	0.67	(0.23 – 1.91)	.45	Moroccan	1.89	(0.71 – 5.03)	.20
Low HDL cholesterol	Dutch	1.71	(0.72 – 4.07)	.23	Dutch	0.99	(0.47 – 2.07)	.98
	Turkish	0.74	(0.38 – 1.41)	.35	Turkish	1.46	(0.82 – 2.62)	.20
	Moroccan	0.87	(0.43 – 1.77)	.70	Moroccan	1.09	(0.55 – 2.17)	.80
Diabetes	Dutch	2.51	(0.67 – 9.46)	.17	Dutch	1.24	(0.24 – 6.37)	.80
	Turkish	0.34	(0.09 – 1.22)	.10	Turkish	2.05	(0.71 – 5.90)	.18
	Moroccan	1.15	(0.50 – 2.69)	.74	Moroccan	0.54	(0.19 – 1.49)	.23

Note:

a) odds ratios adjusted for age.

## Discussion

In this study, we found that obesity was associated with the presence of psychological distress, but only in native Dutch women. Hypertension was associated with less psychological distress in Turkish men and more psychological distress in Dutch women. Abdominal obesity, hypercholesterolemia, low HDL cholesterol and diabetes were not associated with psychological distress.

Many researchers have described the bidirectional relation between obesity and depression in both cross-sectional and longitudinal studies [10,31], but the possible association between hypertension and depression is less clear-cut, with both positive and negative associations being reported [32,33]. Previous research has described ethnic/racial differences in the association between obesity and depression, but this is the first study to investigate this in minority populations outside the USA. Our results are in line with the findings of Gavin et al. [11], who found that obesity was associated with an elevated risk of major depressive disorder in white women, but not in black, Latin, or Asian women, or men. Others have reported a stronger association between obesity and depression in white men than in black men [12], or in white men and women than in black men and women [13]. Our findings provide additional evidence that the obesity-depression relationship varies by ethnicity. Potential explanations for these ethnic disparities include genetic factors and social or cultural factors which might mediate or moderate the obesity-depression relationship.

A prevailing line of thought is that obese white women feel more dissatisfied with their bodies, ultimately resulting in depression, than do obese black women, but this view is questioned by recent results from the USA [34]. In adolescents in Turkey overweight is related to body dissatisfaction [35]. Young adults of Turkish or Moroccan origin in the Netherlands also show a preference for a thin body size [36]. Another explanation for ethnic differences in the association between obesity and depression is the challenging, but disputed, hypothesis that disadvantaged groups, like racial/ethnic minority groups, engage in poor health behaviors, such as overeating, as an effective coping mechanism to alleviate psychological distress [37,38]. Alternatively, the association between depression (and subsequently reduced physical activity and increased caloric intake) and obesity will be attenuated in sociodemographic groups with high rates of obesity [13]. Indeed, in our sample only 19.8% of Dutch women were obese, whereas 48.0% of Turkish-Dutch and 48.2% of Moroccan-Dutch women were. From this perspective, ethnic differences in the obesity-depression association may be an artifact of the ethnic differences in the prevalence of obesity.

In this study abdominal obesity was not associated with psychological distress in any of the gender/ethnic subgroups. The IDF criteria that we applied in this study set the cutoffs for men and women so low that more than 50% of the total sample were classified with abdominal obesity [29]. We re-analyzed the data with waist circumference as a continuous predictor variable in logistic regression analyses. Again, the results showed no association between waist circumference and psychological distress, except for Dutch women, in whom a positive association was present (OR 1.03 (95% CI 1.00 – 1.05) p = .02). After controlling for BMI, this association was no longer significant, indicating that abdominal obesity is not necessarily a better measure of the risk associated with depression than overall obesity. The absence of an association between waist circumference and psychological distress is all the more remarkable, as visceral fat accumulation is thought to play a central role in the vicious circle of depression, metabolic syndrome and cardiovascular disease [3,4].

The significant positive association between hypertension and psychological distress in native Dutch women and the significant negative association in Turkish men in our sample was unexpected. Longitudinal studies, also in multi-ethnic cohorts, have not reported a clinically important association between depressive symptoms and hypertension after adjustment for other cardiovascular risk factors [39]. In our sample, controlling for obesity did not attenuate the association between hypertension and psychological distress (results available from the first author).

Some limitations of our study merit discussion. It was a cross-sectional study, so we cannot comment on causal pathways. In addition, we may have underestimated the association between cardiovascular risk factors and psychological distress, because we used the K10, a short self-report screening instrument for depressive and anxiety disorders. Luppino et al. recently showed in a meta-analysis that obesity had a stronger association with clinical depression than with self-reported depressive symptoms. Furthermore, cardiovascular risk factors, amongst which waist circumference, have been show to be specifically associated with somatic-affective depressive symptom clusters [40,41]. By using the short K10, we were not able to differentiate between depressive symptom clusters. In our assessment of the lipid disorders we were restricted by the fact that obtaining fasting blood samples was not feasible in this general health survey. Therefore we were able to describe the HDL cholesterol status, but the other separate lipid disorders could not be distinguished. Another potential source of bias could have resulted from the relatively low response rate (45%). An estimated 7.5-15% of the non-response is due to incorrect residential information in the municipal population register [42]. The available data on the non-responders are insufficient to predict in which way they are biased. However, we have no reason to assume that potential biases might alter our conclusions on the association between the cardiovascular risk factors and psychological distress that were focus of this study. Lastly, the power of our sample was limited, so we may have underestimated the effect of ethnic interactions.

## Conclusion

Many studies have described a positive association between obesity and psychological symptoms. In this population study among natives and immigrants in the Netherlands we found this association only in native Dutch women, but not in Turkish-Dutch or Moroccan-Dutch women, or in men. Abdominal obesity, hypercholesterolemia, low HDL cholesterol level and diabetes were not associated with psychological distress. Hypertension was negatively associated with psychological distress Turkish-Dutch men and positively in native Dutch women. This study shows that the prevention of obesity and depression warrants an ethnically diverse policy. In native Dutch women an integrated approach seems warranted, whereas in Turkish-Dutch and Moroccan-Dutch women obesity and psychological distress may be targeted separately.

## Competing interests

The authors declare that they have no competing interests.

## Authors’ contributions

ACS, JKU and MASW formulated the research question. MASW, APV, JD and ATFB participated in the design of the study. ACS performed the statistical analyses, assisted by JKU and MASW. ACS drafted the manuscript. All authors contributed to the manuscript with their interpretation of results and comments on earlier drafts. All authors read and approved the final manuscript.

## Pre-publication history

The pre-publication history for this paper can be accessed here:

http://www.biomedcentral.com/1471-2458/12/1090/prepub
